# Pitfalls of continuous drug administration methods in pediatric anesthesia to reduce medication errors

**DOI:** 10.1186/s40981-023-00685-x

**Published:** 2023-12-20

**Authors:** Keisuke Yoshida, Yuko Nakano, Satoki Inoue

**Affiliations:** Department of Anesthesiology, Fukushima Medical University, 1, Hikariga-Oka, Fukushima City, Fukushima 960-1295 Japan

**To the editor,**

In pediatric anesthesia, medication errors are particularly likely to occur [1]. The most common medication error in pediatric anesthesia is incorrect dosing, 26% of incorrect doses are because of dilution errors [2]. Various drugs are administered continuously with infusion pumps, and there are generally two methods of administration. The first is to administer drugs with adjusted concentrations, that is, diluted drug solutions based on the patient’s body weight. The second is to administer drugs at a flow rate adjusted based on the patient’s body weight, at a fixed concentration. Since no studies have directly compared the advantage of either method, the choice is typically determined by institutional policies or the drug administered.

The strength of the drug concentration adjustment method is that the flow rate corresponds to dosage (e.g., 1.0 ml/h = 0.2 mcg/kg/min regardless of body weight), enabling seamless management during anesthesia and postoperative intensive care. However, the calculation and dispensing of drugs can be complicated, leading to the risk of dilution error [3]. The pitfall of this method is that errors in drug preparation are likely to go unnoticed at the time of administration.

In contrast, the flow rate adjustment method is a simpler method in terms of both calculation and dispensing in drug preparation. This method is commonly used in the anesthetic management of adults, and serious dose errors are unlikely to occur in pediatric anesthesia even if medical staff are inexperienced in this area (e.g., 0.8 ml/h for 6 kg infants, 8.0 ml/h for 60 kg adults). In addition, the dilution process can be omitted if ready-prepared syringes are available [1, 4], although the flow rate may not be suitable for pediatric patients. The pitfall of this method is that in case of extremely low birth weight infants, it is possible to make the mistake of giving a 10-fold higher dose of drug (e.g., administering a 600 g neonate the appropriate dose of drug for a 6 kg infant).

Although there are multiple factors involved in medication error, one way to address it is that being aware of the pitfalls of each method regardless of which method is used. In addition, countermeasures unique to each facility, such as using a weight-based quick reference chart or routine checks by a pharmacist familiar with pediatric anesthesia, can contribute to medical safety. Further research is necessary to determine which method of drug administration is safer in pediatric anesthesia, taking into account cost and facility proficiency of pediatric anesthesia.

## Data Availability

Not applicable.
